# Consistency between optical coherence tomography and humphrey visual field for evaluating glaucomatous defects in high myopic eyes

**DOI:** 10.1186/s12886-020-01724-2

**Published:** 2020-11-20

**Authors:** Wen Wen, Yuqiu Zhang, Ting Zhang, Xinghuai Sun

**Affiliations:** 1 Department of Ophthalmology & Visual Science, Eye & ENT Hospital, Shanghai Medical College, Fudan University, Shanghai, China; 2 State Key Laboratory of Medical Neurobiology, Institutes of Brain Science, Fudan University, Shanghai, China; 3 Key Laboratory of Myopia, Ministry of Health, Fudan University, Shanghai, China; 4 Shanghai Key Laboratory of Visual Impairment and Restoration, Fudan University, Shanghai, China; 5 Department of Ophthalmology, Lanzhou University No.2 Hospital, Lanzhou, China

**Keywords:** Optical coherence tomography, Humphrey visual field, Agreement, Glaucoma, Myopia

## Abstract

**Background:**

The study is to investigate the influence of high myopia on the consistency between optical coherence tomography (OCT) and visual field in primary open-angle glaucoma (POAG).

**Methods:**

We enrolled 37 patients with POAG with high myopia (POAG-HM group), 27 patients with POAG without high myopia (POAG group), and 29 controls with high myopia (HM group). All subjects underwent Humphrey perimetry (30–2 and 10–2 algorithms). The peripapillary retinal nerve fiber layer (RNFL) and macular ganglion cell-inner plexiform layer (GCIPL) thicknesses were measured using Cirrus HD-OCT. Spearman’s rank correlation analysis was used to determine correlations between OCT and perimetric parameters. Agreement was analyzed by cross-classification and weighted κ statistics.

**Results:**

In POAG group, the cross-classification analysis showed strong agreement between the inferior temporal GCIPL thickness and the mean sensitivity (MS) of 10–2 algorithms (κ = 0.5447, *P* = 0.0048), and good agreement between the superior and inferior RNFL thicknesses and 30–2 MS (κ = 0.4407 and 0.4815; *P* < 0.05). In the POAG-HM group, only the inferior temporal GCIPL thickness showed good agreement with 10–2 MS (κ = 0.3155, *P* = 0.0289) and none of the RNFL sectors were in good agreement with the corresponding MS.

**Conclusions:**

In POAG patients with high myopia, changes in macular measurements were in accordance with visual field defects, and RNFL thickness did not consistently decline with visual field defects due to the effects of high myopia. This study suggests that during diagnosis and follow-up of glaucoma with high myopia, more attention need to be focused on structure and functional defects in macular areas.

## Background

Glaucoma is the second leading cause of blindness worldwide. It is characterized by progressive retinal ganglion cell apoptosis and corresponding visual field defects [1, 2]. To date, optical coherence tomography (OCT) is the most widely used structural imaging technique, and white-on-white perimetry is the gold standard to detect the loss of retinal function due to glaucoma. Because diagnosis and monitoring of glaucoma are based on consistent structural and functional changes, elucidating the relationships between retinal structural damages and visual function defects is essential.

Previous studies used Pearson’s correlation analysis to detect correlations between OCT and perimetric parameters in patients with glaucoma. They showed that global rim area [3] and peripapillary retinal nerve fiber layer (RNFL) thickness are strongly correlated with visual field threshold. However, other studies suggested that RNFL thickness and visual field are not correlated as glaucoma progresses [4–7]. Besides peripapillary parameters, the ganglion cell complex (GCC) and ganglion cell-inner plexiform layer (GCIPL) thicknesses, particularly the inferior and inferior temporal GCIPL regions, show stronger correlations with perimetric parameters [8] than RNFL parameters [9].

Myopia, especially high myopia, was reported to be a risk factor for glaucoma [10]. The prevalence of open-angle glaucoma and suspected glaucoma is two to three times greater in patients with myopia than in individuals without myopia [11]. However, tilt peripapillary atrophy and diffuse retinal thinning, as determined by OCT, and/or visual field abnormalities make it difficult to differentiate glaucomatous damage from myopic changes. Although we previously reported that macular parameters show greater diagnostic potential than RNFL parameters for detecting glaucoma in patients with high myopia [12], the influence of high myopia on the relationship between RNFL thickness or macular structure and visual field defects in patients with glaucoma remains unclear. Therefore, this study aimed to evaluate the correlations and level of agreement between the structural and functional characteristics of patients with glaucoma with or without high myopia to determine the influence of high myopia on the consistency between optical coherence tomography (OCT) and visual field in patients with primary open-angle glaucoma (POAG).

## Methods

### Participants

Three groups of patients, namely, patients with POAG with high myopia (POAG-HM group), patients with POAG without high myopia (POAG group), and patients with high myopia without glaucoma (HM group) were enrolled in our study. Table 1 showed the characteristics of these groups. Glaucoma patients in POAG-HM group and POAG group were not significantly different in in age, best-corrected visual acuity (BCVA) and IOP. Refractive errors and axial length (AL) were not significantly different between POAG-HM and HM group. All patients with glaucoma were recruited from the Glaucoma Clinic of the Eye and ENT Hospital of Fudan University (Shanghai, China). Patients with high myopia without glaucoma were recruited from the Refractive Clinic of the same hospital. The study adhered to the tenets of the Declaration of Helsinki, and all procedures were approved by the human subjects’ review committee of the Eye and ENT Hospital of Fudan University. All participants provided written informed consent before the procedures. All participants included in the study are fully informed about the study and are voluntary for providing data for analysis. All data is recorded and stored in compliance with ethical and data protection guidelines.

**Table 1:** Characteristics of three groups

Variable	HM Mean ± SD	POAG-HM Mean ± SD	POAG Mean ± SD	F	P*	P†	P‡	P§
Age, years	27.34 ± 6.57	37.27 ± 12.04	43.70 ± 14.07	14.911	< 0.001	0.002	< 0.001	0.082
Diopter, D	−6.69 ± 2.77	− 7.51 ± 3.03	− 1.81 ± 1.99	54.025	< 0.001	0.184	< 0.001	< 0.001
BCVA	1.0 ± 0.09	0.9 ± 0.20	0.9 ± 0.22	7.024	0.001	0.004	0.006	0.999
AL, mm	26.71 ± 0.81	26.96 ± 1.32	24.46 ± 1.14	38.940	< 0.001	0.999	< 0.001	< 0.001
IOP, mmHg	15.86 ± 2.75	17.03 ± 4.31	16.83 ± 3.20	1.933	0.151	0.164	0.656	0.999

*HM* patients with high myopia, *POAG-HM* patients with primary open-angle glaucoma and high myopia, *POAG* patients with primary open-angle glaucoma, *SD* standard deviation, *MD* mean deviation, *PSD* pattern standard deviation, *BCVA* best-corrected visual acuity, *AL* axial length

*Analysis of variance for the comparison among the three groups

^†^Bonferroni post hoc test for comparisons between the POAG-HM and HM groups

^‡^Bonferroni post hoc test for comparisons between the POAG and HM groups

^§^Bonferroni post hoc test for comparisons between the POAG-HM and POAG groups

The diagnosis of POAG was based on the presence of glaucomatous optic neuropathy (GON) and a history of elevated intraocular pressure (IOP; ≥ 21 mmHg). GON was identified based on any of the following signs: optic nerve rim thinning, notching, excavation, RNFL defects, or asymmetry in the vertical cup/disc ratio of ≥0.2 between the two eyes. GON was independently assessed by two glaucoma specialists, and inconsistencies between the two specialists were adjudicated by a third glaucoma specialist. Patients were excluded from the study if they had pathologic myopia, media opacities, any other ocular disease, history of ocular surgery or refractive surgery, and systemic diseases, or were using medications that might induce optic neuropathy and all included eyes were phakic. If both eyes in an individual patient satisfied the inclusion criteria, one eye was randomly included in the study. The axial length (AL) of all patients was > 26 mm, and the BCVA was ≥20/25. During the data collection period, the IOPs of patients with glaucoma were all controlled at < 21 mmHg by using antiglaucoma drugs. High myopia was defined as a refractive error < − 6.0D.

All subjects underwent comprehensive ophthalmologic examinations: BCVA, applanation tonometry, digital fundus photography, and Intraocular Lens Master measurement (Carl Zeiss, Jena, Germany). All patients with glaucoma underwent Humphrey perimetry with the 10–2 and 30–2 Swedish interactive thresholding algorithms. Reliable visual field results were defined as ≤33% false positives, false negatives, reliable factor ≤ 15%, and pupil diameter ≥ 3 mm. The total and quadrant mean sensitivity (MS), mean deviation (MD), and pattern standard deviation (PSD) in both 30–2 and 10–2 algorithms were included in subsequent analyses.

All subjects also underwent OCT (Cirrus HD-OCT) data collection. Peripapillary scans were performed on each eye to measure RNFL thickness, and macular scans were performed to measure macular GCIPL thickness. The GCIPL scan summed the thicknesses of the ganglion cell layer and the inner plexiform layer, which represent the ganglion cell bodies and the ganglion cell dendrites, respectively. Only scans with a signal strength of ≥6 and no motion artifacts were kept for analysis. The parameters obtained from the RNFL scans included the average, sector (superior, nasal, inferior, and temporal), and symmetry thicknesses. The parameters obtained from the GCIPL scan included the average, minimum, and sector (superotemporal, superior, superonasal, inferonasal, inferior, and inferotemporal) thicknesses.

According to the structure–function maps developed by Garway-Heath et al. [13] and Kanamori et al. [14], the 10–2 VF test points were divided into four sectors topographically corresponding to GCIPL sectors, and the 52 test points of the 30–2 VF algorithm were divided into four sectors corresponding to RNFL sectors. Based on previous studies [15], the retinal sensitivities of each VF test point expressed in decibels were first converted to apostilbs, averaged for each sector, and converted back to decibels.

### Statistical methods

Age, refractive error, BCVA, IOP, AL, cup/disc ratio, OCT parameters, and perimetric parameters were compared among the three groups using one-way analysis of variance (ANOVA) followed by Bonferroni post hoc test to detect between-group differences. Spearman’s rank correlation coefficient (*r*) was used to determine correlations between OCT and Humphrey perimetric parameters. We assessed concordance between the OCT and perimetric parameters by using the cross-classification method based on quartiles. Agreement was defined as having the same or adjacent quartiles, and disagreement as a difference in one quartile. Extreme disagreement was defined as a difference in two or more quartiles. The level of agreement between the two methods was analyzed using weighted κ statistics. Weighted κ is defined as the percent agreement, adjusted for chance, and allows for different levels of agreement. A weighted κ of 1 represents perfect agreement, so values close to 1 indicate high levels of agreement. κ values of ≥0.75, 0.4–0.75, and < 0.4 were defined as high, moderate, and low agreement, respectively. A *P* value < 0.05 indicates that the agreement between the two methods was statistically significant.

## Results

Thirty-seven patients with POAG and high myopia (POAG-HM group), 29 patients with high myopia (HM group), and 27 patients with POAG without high myopia (POAG group) were enrolled in our study.

Table 2 shows the comparison of perimetric parameters among the three groups. ANOVA revealed significant differences in all of the parameters among the three groups (all *P* < 0.05). Bonferroni post hoc test showed that the MD and PSD values determined using the 30–2 and 10–2 algorithms were significantly smaller in the POAG-HM and POAG groups than in the HM group (*P* < 0.05). However, no significant differences were found in these parameters between the POAG-HM and POAG groups (*P* > 0.05).

**Table 2:** Perimetric parameters

Variable	HM Mean ± SD	POAG-HM Mean ± SD	POAG Mean ± SD	F	P*	P†	P‡	P§
MD in 30–2 program	− 1.49 ± 1.18	−6.92 ± 6.47	−5.30 ± 4.96	10.073	< 0.001	< 0.001	0.016	0.607
MD in 10–2 program	−0.91 ± 1.17	−5.83 ± 6.40	−4.60 ± 4.61	8.584	< 0.001	< 0.001	0.020	0.999
PSD in 30–2 program	2.52 ± 1.44	6.70 ± 4.74	4.98 ± 3.84	10.264	< 0.001	< 0.001	0.049	0.224
PSD in 10–2 program	1.07 ± 0.16	5.39 ± 4.33	3.38 ± 3.31	13.497	< 0.001	< 0.001	0.037	0.075

*HM* patients with high myopia, *POAG-HM* patients with primary open-angle glaucoma and high myopia, *POAG* patients with primary open-angle glaucoma, *SD* standard deviation, *MD* mean deviation, *PSD* pattern standard deviation

*Analysis of variance for the comparison among the three groups

^†^Bonferroni post hoc test for comparisons between the POAG-HM and HM groups

^‡^Bonferroni post hoc test for comparisons between the POAG and HM groups

^§^Bonferroni post hoc test for comparisons between the POAG-HM and POAG groups

Table 3 lists the OCT parameters in the three groups. ANOVA revealed significant differences among the three groups in the average RNFL thickness and RNFL thicknesses in all sectors, except for the nasal RNFL. The post hoc test showed that the RNFL was significantly thinner in the POAG-HM and POAG groups than in the HM group for the average thickness and RNFL thicknesses in all sectors, except for the nasal sector. However, no significant differences were found in any of the RNFL parameters between the POAG-HM and POAG groups.

**Table 3:** Optical coherence tomography parameters

Variable	HM (*N* = 29)	POAG-HM (*N* = 37)	POAG (*N* = 27)	F	P*	P1^†^	P2^‡^	P3^§^
**RNFLT,** μm	Mean ± SD	Mean ± SD	Mean ± SD					
Average	92.46 ± 10.46	72.03 ± 13.95	77.96 ± 18.18	14.446	< 0.001	< 0.001	0.001	0.990
Superior	107.89 ± 13.72	83.22 ± 17.84	91.46 ± 26.58	12.936	< 0.001	< 0.001	0.011	0.239
Nasal	63.89 ± 11.14	67.16 ± 13.51	67.08 ± 9.46	0.083	0.920	0.999	0.999	0.999
Inferior	109.64 ± 19.23	75.05 ± 20.88	89.85 ± 31.69	12.104	< 0.001	< 0.001	0.011	0.337
Temporal	88.82 ± 17.13	62.78 ± 18.68	63.81 ± 19.10	17.050	< 0.001	< 0.001	< 0.001	0.999
**GCIPLT, μm**								
Average	79.21 ± 4.27	62.06 ± 11.10	71.88 ± 10.37	19.483	< 0.001	< 0.001	< 0.001	0.014
Minimum	77.75 ± 4.70	51.77 ± 12.80	65.00 ± 12.22	34.468	< 0.001	< 0.001	< 0.001	< 0.001
Superotemporal	79.50 ± 4.12	61.83 ± 13.80	71.08 ± 10.54	16.403	< 0.001	< 0.001	0.005	0.096
Superior	80.54 ± 4.83	64.06 ± 13.50	73.31 ± 12.13	11.100	< 0.001	< 0.001	0.086	0.077
Superonasal	81.21 ± 5.48	65.34 ± 15.57	77.38 ± 11.52	9.920	< 0.001	< 0.001	0.814	0.010
Inferonasal	78.64 ± 4.52	63.17 ± 12.24	73.31 ± 10.94	9.642	< 0.001	< 0.001	0.303	0.043
Inferior	75.75 ± 5.34	57.17 ± 10.09	68.15 ± 10.91	23.214	< 0.001	< 0.001	0.008	0.003
Inferotemporal	79.50 ± 3.95	58.51 ± 10.64	67.92 ± 11.95	31.606	< 0.001	< 0.001	< 0.001	0.016

*HM* patients with high myopia, *POAG-HM* patients with primary open-angle glaucoma and high myopia, *POAG* patients with primary open-angle glaucoma, *RNFLT* retinal nerve fiber layer thickness, *SD* standard deviation, *GCIPLT* ganglion cell-inner plexiform layer thickness

*Analysis of variance for the comparison among the three groups

^†^Bonferroni post hoc test for comparisons between the POAG-HM and HM groups

^‡^Bonferroni post hoc test for comparisons between the POAG and HM groups

^§^Bonferroni post hoc test for comparisons between the POAG-HM and POAG groups

ANOVA revealed significant differences among the three groups in all GCIPL thicknesses, including the average, minimum, and all sectorial GCIPL. The GCIPL was significantly thinner in the POAG-HM group than in the HM group. The average, minimum, superotemporal, inferior, and inferotemporal thicknesses were significantly greater in the HM group than in the POAG group (all *P* < 0.05), but no significant differences were found in the superior, superonasal, and inferonasal sectors between these groups (*P* > 0.05). In addition, the average, minimum, superonasal, inferonasal, and inferior GCIPL regions were significantly thinner in the POAG-HM group than in the POAG group (*P* < 0.05), but no differences were noted in the superotemporal and superior GCIPL thicknesses between the two groups (*P* > 0.05).

Tables 4, 5 and 6 show the correlations and agreement between OCT and perimetric parameters in the HM, POAG-HM, and POAG groups, respectively. In the HM group (Table 4), none of the MS sectors determined using the 10–2 algorithm was correlated with its corresponding GCIPL (*r* = 0.3125–0.1102, *P* > 0.05). Furthermore, none of the RNFL sectors was correlated with the corresponding MS determined using the 30–2 algorithm (*r* = 0.0538–0.3077, *P* > 0.05), except for the nasal sector. The cross-classification analysis yielded similar results, in that the GCIPL and RNFL parameters showed low consistency with the corresponding MS, with weighted κ values ranging from − 0.2656 to 0.3061 (*P* > 0.05).

**Table 4:** Correlation and agreement between optical coherence tomography and perimetric parameters in patients with high myopia

	Spearman’s rank correlation		Cross-classification (%)		Kappa analysis		
	r	p	Same quartile	Adjacent quartile	Opposite quartile	Weight kappa	*P* value
**10–2 SITA VS GCIPL**							
Superior temporal MS VS Inferior nasal GCIPL	0.0474	0.8144	26	63	11	0.0505	0.9209
Superior nasal MS VS Inferior temporal GCIPL	−0.0362	0.8577	11	70	19	−0.1765	0.0926
Inferior temporal MS VS Superior nasal GCIPL	0.1102	0.5843	41	44	15	0.2041	0.0647
Inferior nasal MS VS Superior temporal GCIPL	−0.3125	0.1125	15	74	11	−0.1898	0.2144
**30–2 SITA VS RNFL**							
Superior MS VS Inferior RNFL	0.3077	0.1184	37	52	11	0.3061	0.1514
Nasal MS VS Temporal RNFL	−0.0538	0.7898	22	63	15	−0.0658	0.7415
Inferior MS VS Superior RNFL	0.0138	0.9457	15	70	15	−0.0848	0.2170
Temporal MS VS Nasal RNFL	−0.4100	0.0337*	15	63	22	−0.2656	0.2170

*SITA* Swedish interactive thresholding algorithms, *GCIPL* ganglion cell-inner plexiform layer, *MS* mean sensitivity, *RNFL* retinal nerve fiber layer

* statistically significant, *P* < 0.05

**Table 5:** Correlation and agreement between optical coherence tomography and perimetric parameters in patients with primary open-angle glaucoma with high myopia

	Spearman’s rank correlation		Cross-classification (%)		Kappa analysis		
	r	p	Same quartile	Adjacent quartile	Opposite quartile	Weight kappa	*P* value
**10–2 SITA VS GCIPL**							
Superior temporal MS VS Inferior nasal GCIPL	0.4226	0.0353*	32	64	4	0.2523	0.4259
Superior nasal MS VS Inferior temporal GCIPL	0.4022	0.0462*	44	44	12	0.3155	0.0289*
Inferior temporal MS VS Superior nasal GCIPL	0.1490	0.4773	24	68	8	0.0693	0.8965
Inferior nasal MS VS Superior temporal GCIPL	0.1117	0.5951	36	52	12	0.1199	0.2080
**30–2 SITA VS RNFL**							
Superior MS VS Inferior RNFL	0.5325	0.0025*	33	67	0	0.2703	0.2911
Nasal MS VS Temporal RNFL	−0.2713	0.1471	27	53	20	−0.1517	0.8430
Inferior MS VS Superior RNFL	0.5095	0.0040*	33	60	7	0.2527	0.2983
Temporal MS VS Nasal RNFL	0.1448	0.4453	30	60	10	0.1081	0.5265

*SITA* Swedish interactive thresholding algorithms, *GCIPL* ganglion cell-inner plexiform layer, *MS* mean sensitivity, *RNFL* retinal nerve fiber layer

*  statistically significant, *P *< 0.05

**Table 6:** Correlation and agreement between optical coherence tomography and perimetric parameters in patients with primary open-angle glaucoma

	Spearman’s rank correlation		Cross-classification (%)		Kappa analysis		
	r	p	Same quartile	Adjacent quartile	Opposite quartile	Weight kappa	*P* value
**10–2 SITA VS GCIPL**							
Superior temporal MS VS Inferior nasal GCIPL	0.2722	0.2596	32	63	5	0.1624	0.5198
Superior nasal MS VS Inferior temporal GCIPL	0.7278	0.0004*	53	47	0	0.5447	0.0048*
Inferior temporal MS VS Superior nasal GCIPL	0.3118	0.1937	37	47	16	0.2262	0.2269
Inferior nasal MS VS Superior temporal GCIPL	0.6365	0.0034*	32	68	0	0.3567	0.5017
**30–2 SITA VS RNFL**							
Superior MS VS Inferior RNFL	0.7132	0.0002*	45	55	0	0.4815	0.0263*
Nasal MS VS Temporal RNFL	0.3056	0.1666	32	68	0	0.1987	0.4739
Inferior MS VS Superior RNFL	0.6102	0.0026*	45	55	0	0.4407	0.0279*
Temporal MS VS Nasal RNFL	0.1218	0.5890	23	68	9	−0.0034	0.7705

*SITA* Swedish interactive thresholding algorithms, *GCIPL* ganglion cell-inner plexiform layer, *MS* mean sensitivity, *RNFL* retinal nerve fiber layer

* statistically significant, *P *< 0.05

In the POAG group (Table 6), the inferior temporal and superior temporal GCIPL thicknesses were positively correlated with the corresponding MS determined using the 10–2 algorithm (*r* = 0.6365, *P* = 0.0034; *r* = 0.7278, *P* = 0.0004). The superior and inferior RNFL thicknesses in the POAG group were strongly correlated with the corresponding MS determined using the 30–2 algorithm (*r* = 0.6102, *P* = 0.0026; *r* = 0.7134, *P* = 0.0002). In the cross-classification analysis, the superior and inferior RNFL thicknesses in the POAG group showed moderate agreement with the corresponding MS determined using the 30–2 algorithm (κ = 0.4407, *P* = 0.0279; κ = 0.4815, *P* = 0263). In addition, the inferior temporal GCIPL thickness showed moderate agreement with the corresponding MS (κ = 0.5447, *P* = 0.0048) in the POAG group.

In the POAG-HM group (Table 5), the GCIPL thicknesses of the inferior nasal and inferior temporal sectors were positively correlated with the corresponding MS determined using the 10–2 algorithm (*r* = 0.4226, *P* = 0.0353; *r* = 0.4022, *P* = 0.0462). Similar to the POAG group, the superior and inferior RNFL thicknesses were highly correlated with the corresponding MS determined using the 30–2 algorithm (*r* = 0.5095, *P* = 0.0040; *r* = 0.5325, *P* = 0.0025). In the cross-classification analysis of OCT and perimetric parameters, only the inferior temporal GCIPL thickness showed significant agreement with the corresponding MS determined using the 10–2 algorithm (κ = 0.3155, *P* = 0.0289) and none of the RNFL sectors were in good agreement with the corresponding MS.

## Discussion

In this study, we evaluated the relationship between OCT and perimetric defects in patients with POAG with or without high myopia to investigate the influence of high myopia on structural and functional evaluation of glaucomatous defects. We also used these data to evaluate the level of consistency between structural and functional parameters.

RNFL thickness and visual field defects are widely used by ophthalmologists to evaluate GON. The most commonly used tools to assess structure and function are OCT and perimetry, respectively. Patients with high myopia usually have a distorted optic disc, peripapillary atrophy, and posterior scleral staphyloma, which may interfere with the accuracy of RNFL thicknesses measured by OCT and glaucoma severity evaluation, as assessed by ophthalmologists. Long axial length is proven to affect the accuracy of macular thickness maps in OCT scan and in normal subjects the strong correlation between the thickness of inner retinal layers and axial length appared to result from magnification effects. Hence we can’t distinguish that the declined thickness of inner retinal layers (including RNFL and GCIPL) in glaucoma patients with high myopia is due to increasing axial lengths or glaucomatous defects [16, 17]. Additional low concordance to the rules of inferior>superior>nasal>temporal (ISNT) was observed in myopia (e.g., for RNFL, 8% of high axial myopes compared with 67% of emmetropes [18]). Furthermore, visual field examination of patients with high myopia often shows declining visual sensitivity and/or local visual field loss, which would also disturb the judgment of glaucomatous visual field defects. For these reasons, investigating the relationship between structural and functional defects is necessary in patients with glaucoma with or without high myopia to improve the clinical evaluation and follow-up of these patients.

Both macular GCC and average RNFL thickness negatively correlate with AL, indicating high myopia would cause thinning of GCC and RNFL [19]. Since the inferotemporal and superotemporal RNFL bundles tend to converge temporally with raising myopia [20] and some OCT do not have a normal database for eyes with high myopia, OCT measurements in patients with high myopic glaucoma cannot reflect the condition of ganglion cell apoptosis due to glaucoma [21]. Previous studies showed conflicting findings about diagnostic ability of OCT parameters in high myopic glaucoma. Shoji et al. [22, 23], Zhang et al. [12], Wang et al. [19], and Hung et al. [24] showed that GCC parameters are significantly better for detecting high myopic glaucoma than RNFL. However, a study [25] reported no significant difference in the detection ability between RNFL and GCC thicknesses. Thus, distinguishing the glaucomatous defects from high myopia changes only by structural or functional evaluations is difficult. Investigation on structure-function relationship in glaucoma with high myopia is necessary. Studies focusing on this relationship are limited. Shin et al. found that peripapillary vessel density-VF association is stronger than RNFL-VF association [26], indicating that RNFL is not well related to VF in high myopia glaucoma. In our study, we used several methods (Spearman’s correlation coefficients, cross-classification method, and weighted κ) to investigate the concordance between OCT measurements and visual field sensitivity. In HM group, no significant inter-examination relationship and agreement could be found. We think although RNFL or macular thickness decrease or declining visual sensitivity and/or local visual field loss might be observed in high myopia patients, but the agreement between structural abnormity and functional defects are one of the specific clinical features of glaucoma. In the POAG group, the inferior temporal and superior temporal GCIPL were significantly correlated with the corresponding MS determined using the 10–2 algorithm by Spearman’s correlation analysis, which is consistent with a previous report [27]. However, the weighted κ value was significant only for the inferior temporal GCIPL (κ = 0.5447, *P* = 0.0048), which indicates that the inferior temporal GCIPL defects due to glaucoma show the best correlation with the corresponding 10–2 visual field sensitivity. With regard to the concordance between RNFL parameters and visual field sensitivity, the superior and inferior RNFL thicknesses were positively correlated with the corresponding MS determined using the 30–2 algorithm with Spearman’s rank correlation coefficients and cross-classification analysis. In the POAG-HM group, significant correlation coefficients could be found for the inferior nasal GCIPL, inferior temporal GCIPL, inferior RNFL, and superior RNFL. However, the consistency between the two assessments was weaker in the POAG-HM group than in the POAG group because only the inferior temporal GCIPL showed significant consistency with the corresponding MS (κ = 0.3155, *P* = 0.0289). These findings indicate that the visual field defects in patients with glaucoma and high myopia are consistent in macular measurements but not in RNFL thicknesses. These results agree with our belief that macular measurements show significantly better ability than RNFL thickness to detect glaucoma. We speculate that this discrepancy is due to peripapillary atrophy in high myopia that affects the relationship between the structural and functional defects, whereas macula is less likely to be affected by high myopia.

Some studies suggested that the structural and functional changes in the progression of glaucoma do not occur in parallel [4]. However, other studies found moderate to strong relationships between the structural and functional parameters [28, 29]. Kim et al. reported that RNFL and GCC thicknesses show similar structural and functional relationships with the perimetric sensitivity in glaucomatous patients [30]. Meanwhile, Bowd et al. reported that RNFL thicknesses (mean, superior, and inferior) measured using scanning laser polarimetry are significantly associated with their corresponding visual field zones, with R^2^ values ranging from 0.13 to 0.20 [28]. Leung et al. reported that RNFL thicknesses measured using Stratus OCT show strong associations with visual function (R^2^ = 0.623) [29]. We speculate that the variation in the correlation coefficients observed in prior studies is due to the differences in disease severity, instruments used, and analytical parameters. Most previous studies used either Pearson’s correlation coefficient or Spearman’s rank correlation coefficient to describe the correlations between structural and functional parameters. Correlation coefficients represent the strength and direction of a linear relationship between two random variables, but do not represent the level of agreement between variables. Therefore, in our study, we used cross-classification and κ analysis to measure the level of agreement between structural and functional defects.

There were no significant differences in refractory errors and axial lengths between HM group and POAG-HM group. Although patients in HM-group were younger than the other two groups, we think it would not affect our results and conclusion. Firstly, we didn’t compare the structural or functional changes between groups but the agreement within each group. Although previous studies showed retinal structures was reported to be decline with the age, it was proven the RNFL thickness and VF sensitivities declined at similar rates [31], implying the structure-function relationship might be not affected by age. Secondly, the main aim of our study is to investigate the structure-function agreement in POAG patients with or without high myopia, in which two groups there were no significant differences in age.

The limitations of this study included the relatively small sample size and cross-sectional study design. Previous studies showed the agreement between functional and structural examinations tends to decrease as glaucoma progresses. For example, consistency between combined structure function index (CSFI) and standard automated perimetry (SAP) indices in middle-stage glaucoma was reported to be low [32]. Further studies are warranted to examine the consistency between OCT and visual field parameters during progression in glaucoma patients with high myopia.

## Conclusions

For patients with POAG, with or without high myopia, the decline in retinal function occurred along with structural damage. In contrast to patients with POAG without high myopia, we observed consistency between visual field and macular measurements, but not with RNFL thicknesses, in patients with POAG and high myopia. This study suggests that during diagnosis and follow-up of glaucoma with high myopia, more attention need to be focused on structure and functional defects in macular areas.

## Data Availability

The datasets used and/or analysed during the current study are available from the corresponding author on reasonable request.

## Abbreviation

OCT: Optical coherence tomography
POAG: Primary open-angle glaucoma
HM: High myopia
RNFL: Retinal nerve fiber layer
GCIPL: Macular ganglion cell-inner plexiform layer
GCC: The ganglion cell complex
BCVA: Best-corrected visual acuity
GON: Glaucomatous optic neuropathy
MS: Mean sensitivity
MD: Mean deviation
PSD: Pattern standard deviation
VF: Visual field
